# 788. Social media as a powerful tool for expanding infectious disease-related medical knowledge: A Twitter account perspective

**DOI:** 10.1093/ofid/ofad500.849

**Published:** 2023-11-27

**Authors:** Mario A Torres, Richard A Zuckerman, Rebecca Mazurkiewicz

**Affiliations:** Dartmouth Health, Lebanon, New Hampshire; Dartmouth-Hitchcock Medical Center, Lebanon, NH; Nuvance Health - Vassar Brothers Medical Center, Poughkeepsie, New York

## Abstract

**Background:**

The influence of social media on medical education has been demonstrated by multiple meta-analyses, highlighting the important role of this medium in the medical community. Within the realm of available platforms, Twitter plays a major role. The goal of this project was to create an account to propagate medical content and attract learners to the field of Infectious Disease.

**Methods:**

In 2021 @theIDtrivia was founded, an account dedicated to sharing Infectious Disease-related content, creating trivia (Figure 1-A) and “board-style” questions based on the American Board of Internal Medicine Infectious Disease Certification Exam Blueprint. After posting questions in a poll-type format, users have 3-7 days to select an answer before the correct one is posted along with an infographic (Figure 1-B). All tweets and infographics are well-researched, and the references are shared with links embedded with the intent of developing a reliable social media resource.

**Figure:**
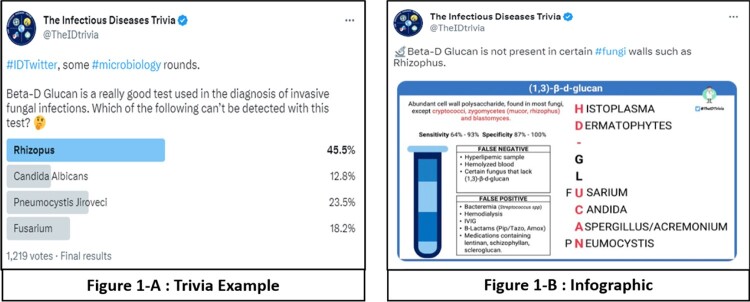

**Results:**

The first tweet was posted on September 2021, and the account had just one follower at the time. Over the next 18 months, 881 tweets have been posted and the account has gained over 7500 followers (Graph 1-A) - 35% are active accounts from the United States (Figure 2). The number of impressions (times a user is served a tweet in their timeline or search results, Graph 1-B) has increased concomitantly with engagement with the account (total number of times a user interacts with a tweet, including retweets, replies, likes). Based on responses, people at multiple stages of their careers, practitioners of other areas such as pharmacy and microbiology, and institutional accounts from different parts of the world have actively engaged with the content of @theIDtrivia.

**Figure:**
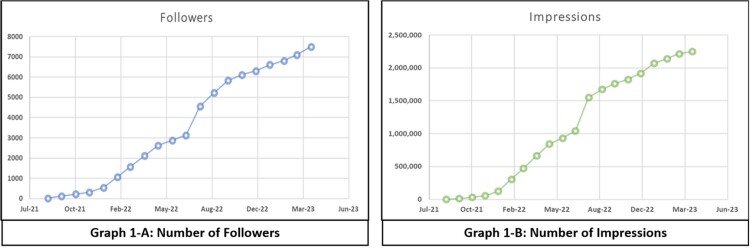

**Figure:**
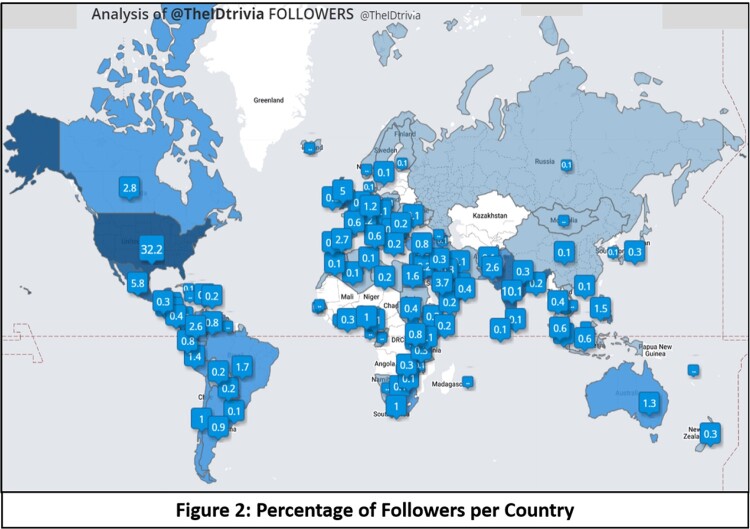

**Conclusion:**

We have shown that infectious disease-based trivia via social media engages a wide and varied population interested in exchanging knowledge and ideas. While this is positive in and of itself, one limitation is that we are unable to show that those who follow the account and/or participate in its content, feel that it results in improved test performance or in clinical practice. This opens an opportunity for continued exploration of the role of social media in medical education and how it can be integrated into clinical decision-making.

**Disclosures:**

**All Authors**: No reported disclosures

